# Design and Validation of an Instrument To Measure a Minor's Maturity When Faced with Health Decisions

**DOI:** 10.1007/s11673-019-09930-4

**Published:** 2019-08-01

**Authors:** Eva Miquel, Montserrat Esquerda, Jordi Real, Mariola Espejo, Josep Pifarré

**Affiliations:** 1 Universitat de Lleida (Udl) – IRBLleida, Lleida, Spain; 2 Institut Català de la Salut, Lleida, Spain; 3 Institut Borja de Bioética, Universitat Ramon Llull, C/Santa Rosa 6, Esplugues, Barcelona, Spain; 4 Sant Joan de Déu Terres de Lleida, Lleida, Spain; 5 Unitat de Suport a la Recerca Lleida, Institut Universitari d’Investigació en Atenció Primària Jordi Gol (IDIAP Jordi Gol), Lleida, Spain; 6 Hospital Universitari Santa Maria, Lleida, Spain

**Keywords:** Mature minor, Adolescent, Capacity, Health decision-making, Ethics

## Abstract

Decision-making capacity in children and adolescents in healthcare requires thorough assessment: the minor's maturity, understanding of the decision, risk of the situation and contextual factors needs to be explored. The intention was to design and validate a test—the Maturtest—to assess the maturity of minors in decision-making processes in healthcare. A reasoning test on moral conflicts for adolescents was designed to infer the degree of maturity of minors applied to decision-making regarding their own health. The test was completed by a sample of 441 adolescents aged from twelve to sixteen, with a corresponding analysis of their psychometric skills to measure feasibility, viability, reliability, validity, and sensitivity to change. Psychometric test results showed viability, reliability, validity, and sensitivity to change. High correlation (correlation index = 0.74) between the test score and the reference method were notable. A high stability was obtained with an intraclass correlation coefficient (r = 0.77). The average response time of the test was twenty-three minutes. This test measures the moral maturity of adolescents. It is presented as an objective, useful, valid, reliable tool, easy to fill out, edit and apply in a healthcare context. It helps to assess the maturity of minors faced with a decision.

## Background

Since the second half of the twentieth century, a profound paradigm shift has changed the healthcare of minors, moving from a model based on paternalism towards a new model, in which the child is not only the object of protection but also a subject of rights, proportional to their degree of maturity and development (Streuli, Michel, and Vayena 2011; Beauchamp and Childress 1994). Because this paradigm shift has a direct impact on healthcare practice and on research, clinicians must be trained to assess the ability of a minor to make a correct health decision. This assessment should not be considered a one-time event and represents an important challenge, because of the lack of a systematic assessment and standardized procedures (Hein et al. 2015; Ruhe et al. 2015).

There are different approaches to assessing a minor’s competence. People are considered competent to decide if they can understand the information they receive and take full responsibility for the corresponding decision in accordance with their personal values (Colby and Kolberg 1987). Colby and Kolberg (1987) defines competence as the ability to make decisions and judgments that are based on internal principles and to act in accordance with these judgments.

Regarding the assessment of the minor's maturity, studies by Piaget, Kolhberg (1987), and other authors on the development of autonomy and moral and ethical development support the theory of the “mature minor,” according to which most adolescents reach their moral maturity between thirteen and fifteen years of age.

Because of the “mature minor theory,” laws in different countries have changed, both concerning the acquisition of legal majority and the progressive ability to directly exercise some of these rights prior to reaching that age, including the right to take healthcare-related decisions (Stultiens et al. 2007). It is important to remark that the basis of the development of the “mature minor’s theory” are the moral development studies.

Colby and Kolberg (1987) recommends that the development of moral maturity should go through several stages (Table 1), each of which represents a qualitative difference from previous stages. For Kohlberg, 80 percent of children are in a preconventional stage until an age of ten to twelve, the conventional stage is that of adults, while the postconventional stage is restricted to a small group of persons.

**Table 1:** Moral development levels and stages according to Kohlberg

Moral development level	*Stage*
**Preconventional.** Individualistic concrete perspective, centered in onesellf. Imposed external rules.	**I. Heteronomous moral** Punishment-obedience direction and social egocentric perspective **II. Individualism, instrumental purpose and exchange** Istrumental-relativistic direction and social individualistic concrete perspective
**Conventional** Member of society perspective. Identification of individual with rules	**III. Relations, mutual interpersonal expectations and interpersonal conformity** Concordance interpersonal direction and social individual perspective, when relations with others. **IV. Social system and conscience** Legalist and authoritarian direction and social perspective of diferentiation of different points of view about agreement or interpersonal reasons.
**Postconventional** “Presceeding” to society perspective, about principles moral reasoning. Indiviual distinguish between rules and his own values.	**V. Contract or social uesfulness and individual rights** Social contract direction – upper principles and social perspective previous to society. **VI. Universal ethic principles** Social perspective consist on recognition of universal moral principles from which social commitments derive because human being is a purpose in itself so it must be recognised.

Most instruments for measuring moral maturity of adolescents have been designed and applied in highly complex educational and research environments. They are not suited to a clinical practice environment (Hein et al. 2015; McCabe et al. 1996; Weithorn and Campbell 1982). It is therefore crucial to develop an assessment or screening tool for the maturity of minors that can be easily corrected and applied within the normal time frame of a clinical consultation.

In the face of the challenge posed in assessing the competence of a minor, this study examines the possibility of assessing the maturity of a minor within a clinical context.

## Method

An observational and interdisciplinary design of a test was performed, with a sample of 441 adolescents from Lleida from twelve to sixteen years old and a subsample of 76 individuals in the second phase (seven days later).

### Measures

#### Main Variables

The validation test (called Maturtest, see Appendix 1) is a self-administered questionnaire consisting of seventeen closed questions with hypothetical situations that give rise to a moral dilemma. In each case, there are two preconventional answers and two conventional answers, based on the Kohlberg level of moral maturity (Thurstone 1972). A preliminary scale (Espejo et al. 2011) of attitude measurement was created based on the Thurstone scale model. The questions on judgment were designed starting from the subgroups described in Colby and Kolberg (1987), where a whole multidisciplinary team of experts in the subject participated (a psychiatrist, a pediatrician, a surgeon, a family doctor, a psychologist, a philosopher, and a primary care nurse) in order to study the construct validity of each of the resulting questions. The method used is the so-called “expert judgement criteria.”

The moral dilemmas adapted from classic dilemmas were elaborated and the answers were classified according to the Kohlberg model into two groups: preconventional response or conventional responses (postconventionality is not usual in adolescents). Independently and blindly, the experts classified the stage of each response, as well as validating the questions. The classification of each one of the answers was made according to the degree to which each one of them measured a determined degree of maturity, not depending on the attitude expressed by the expert. The cases where there was discrepancy of opinions were eliminated or modified until unanimity of assessment was obtained, so that in the final eighteen cases there was a consensus among all the experts and an objective evaluation was made of the degree to which each of the answers measured a certain degree of maturity.

This structured questionnaire consists of seventeen hypothetical situations that give rise to a moral dilemma. The adolescents had to choose which of four responses to each case they considered more appropriate. The answers correspond to two preconventional and two conventional responses, according to the levels of moral maturity of Kolhberg and based on the criterion of expert judgment. It computes the number of mature responses within a range of values between 0 and 17.

The Gold-standard is the Moral Judgement Interview (MJI) (Colby and Kolberg 1987). This is a semi qualitative interview with open questions about moral dilemmas (ratings range from 100 to 400). The interview requires trained personnel for both administration and correction. The evaluation of the tutor is done with a Likert scale, assessing maturity, cognitive development and academic achievement of their students on a subjective basis (with ratings range from 1 to 5). Secondary variables are gender, origin, age, year, participating centre, time spent performing the test.

### Data Analysis

The descriptive variables of the study are reported in table 2. The study follows an evaluation procedure of instruments for measuring quality of life related to health based on the proposal from the IRYSS network (Valderas, Ferrer, and Alonso 2005), the Scientific Committee of the Medical Outcomes Trust (2002), Guyatt (1993), and Hays (1993). The performance of four key attributes is assessed: 1. Feasibility/viability, that is, ease of use of the instrument, 2. Reliability or stability of results in similar circumstances (internal consistency, correlation coefficient between the test items, reliability of test-retest, consistency of two measurements based on the same questionnaire), 3. Validity: the degree to which the instrument reflects what it is intended to measure (content, structure, “ceiling effect” and “floor effect,” construct, criteria), 4. Sensitivity to change: or ability of an instrument to detect changes in the attribute of interest due to an intervention or to the natural evolution of the illness (“effect size”) (tables 3, 4, and 5).

**Table 2:** Population characteristics

	Number	Percentage
Gender		
Boy	219	50%
Girl	219	50%
Age (years)		
12	47	10.7%
13	95	21.7%
14	93	21.2%
15	151	34.5%
16	52	11.9%
Kind of school		
Public	202	46.1%
Private	236	53.9%

**Table 3:** Psychometric characteristics and T17 and T9 correlations with MJI weighted average score, teacher's value (convergent/divergent validity) and retest

Psychometric characteristics	T-17	T-9
Item number	17	9
Theoretical rank	0-17	0-9
Observed rank	3-17	0-9
KR-20	0.43	0.46
Ceiling effect N (%)1	11(2.5)	48(11)
Floor effect N (%)2	1(0.2)	1(0.2)
Average (dt)	13.0 (2.1)	6.4 (1.7)
Correlation coefficient with the test (3):		
Gold Standard		
MJI weighted average score	0.74	0.65
Teacher's value		
Maturity	0.17	0.16
Cognition	0.21	0.15
Academics	0.18	0.17
Retest	0.79	0.77

1: Cases frequency with maximum punctuation; 2: Cases frequency with minimum punctuation; 3: Statistical significative correlations (p value<0.01)

**Table 4:** Extreme groups validity: differences between punctuations of the test in different groups

			IC95%				Effect	
Group		Average	(L inf	- Lsup)	Minimum	Maximum	size	valor p
Gender		Test T-17						
Boy		12.7	(12.4-	13.0)	3	17	-0.41	<0.001*
Girl		13.4	(13.2-	13.7)	6	17		
		Test T-9						
Boy		6.1	(5.8-	6.3)	1	9	-0.41	<0.001*
Girl		6.7	(6.5-	6.9)	0	9		
Age in years		Test T-17						
12		13.5	(12.9-	14.1)	9	17	-	0.307
13		13.1	(12.8-	13.6)	7	17	-0.16	
14		12.8	(12.3-	13.3)	6	17	-0.34	
15		12.9	(12.6-	13.2)	3	17	-0.3	
16		13.2	(12.6-	13.8)	6	17	-0.14	
		Test T-9						
12		7.0	(6.5-	7.5)	2	9	-	0.072
13		6.5	(6.1-	6.9)	1	9	-0.29	
14		6.2	(5.8-	6.6)	0	9	-0.46	
15		6.3	(6.0-	6.5)	1	9	-0.4	
16		6.4	(5.9-	6.9)	2	9	-0.32	
Kind of school		Test T-17						
Private		12.9	(12.7-	13.2)	6	17	-0.12	0.207
Public		13.2	(12.9-	13.5)	3	17		
		Test T-9						
Private		6.3	(6.0-	6.5)	1	9	-0.19	0.05*
Public		6.6	(6.3-	6.8)	0	9		

*Statistical significance level 5%; CI 95: Confidence Interval 95%

**Table 5:** Sensibility to change evaluation (effect size)

		CI95%				Effect	
Test	Average	(L inf	- Lsup)	Minimum	Maximum	Size	P value
T17							
Test	13.04	(12.8-	13.23)	3	17	-0.24	0.045*
Retest	13.43	(12.8-	14.04)	4	17		
T9							
Test	6.51	(6.24-	6.56)	0	9	-0.37	0.002*
Retest	7.00	(6.58-	7.37)	2	9		

*p value<0.05 resulting from t-student test for suitable samples

Each participant was classified into one of three categories of possible maturity according to the Maturtest. Each of these is associated with different stages of maturity as postulated by Kohlberg.

The psychometric analysis was performed simultaneously, based on the initial test of seventeen items (T-17) and a reduced version of nine items (T-9). The reduced version of the test was obtained through a reliability analysis.

## Ethical Considerations

This study was approved by the Comitè Ètic d’Investigació Clínica (CEIC) (Clinical Research Ethics Committee) of the Hospital Arnau de Vilanova in Lleida. The study was also granted authorization by the Education Department of the Generalitat de Catalunya and approved by the school directors. The written informed consent was obtained from the parents or guardians on behalf of the minors; the oral assent of the minors involved was also obtained.

## Results

### Descriptive Analysis

The study’s participants were 441 boys and girls aged twelve to sixteen years old from sixteen urban and rural schools in Lleida province. The minors recruited from the schools were randomly selected by conglomerates; three children (1 per cent) were excluded because of invalid or incomplete resolutions of the questionnaires (table 2).

The average number of conventional or mature responses to the test was 13 (range 3–17, SD 2.13) and the average of the scores obtained with the MJI was 235 (range 133–355, SD 55.6). The assessment was conducted on a Likert scale from 1 to 5 and showed an average of 3.5 in cognitive performance, 3.4 in maturity, and 3.2 in intellectual performance (table 3).

### Analysis of the Psychometric Characteristics

**Fig. 1:**
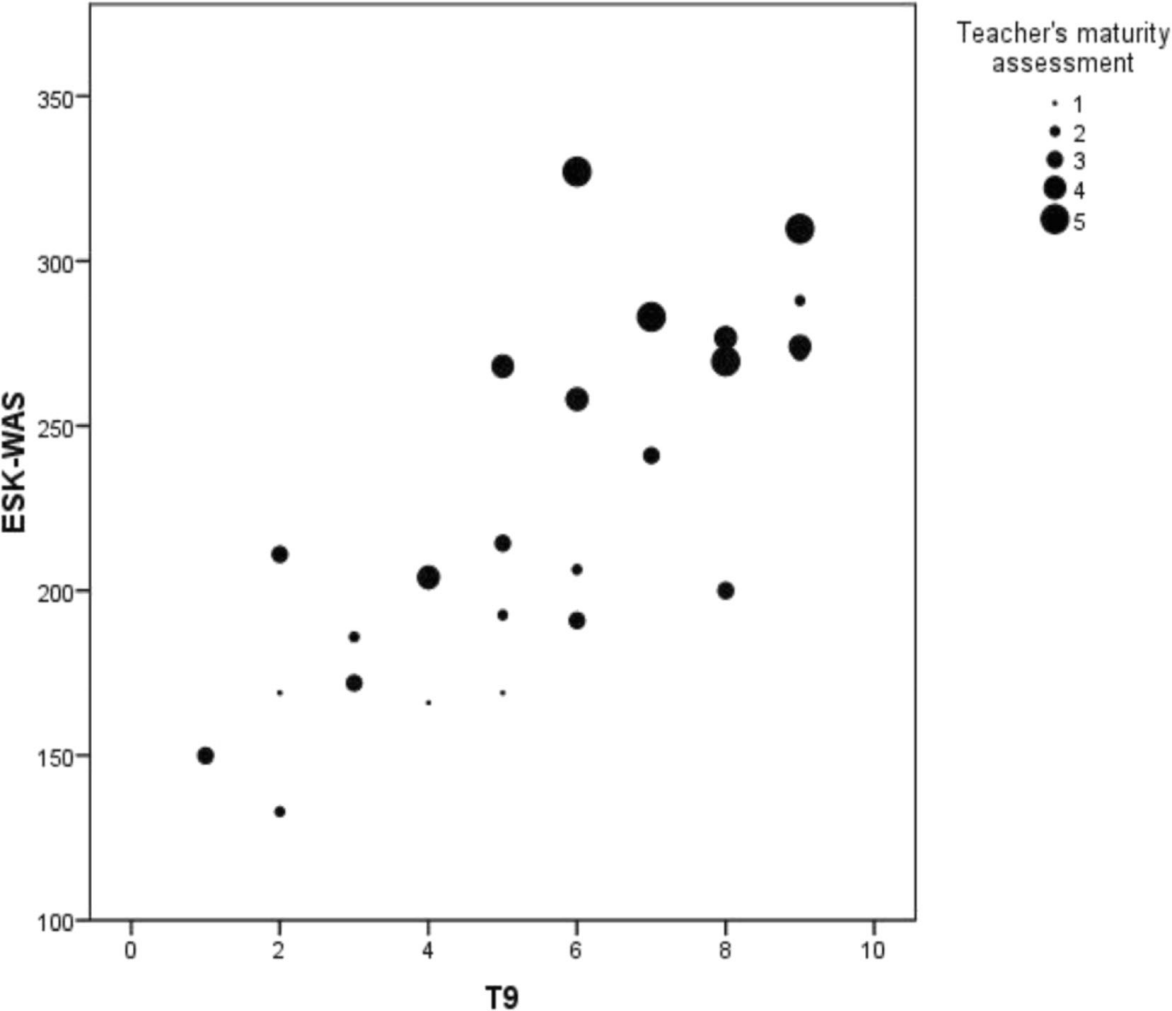
T9 correlation with Kohlberg’s interview (ESK-WAS) and teacher’s maturity assessment

The average time for completion of the test was 23.2 minutes (95 per cent CI: 22.5 to 23.9 minutes); concerning internal consistency, the Kuder Richardson coefficient (KR-20) was 0.43. After downsizing the test to nine items, the coefficient remained at 0.46, with an estimated time of fifteen minutes. Reliability was similar in relation to gender, age, and type of centre. The intraclass correlation coefficient (ICC) of 0.71 showed a positive correlation between the initial and subsequent score. Regarding construct validity, a 0.74 correlation was found between the results of the tests and the results of the Kohlberg MJI (figure 1). We found a significant and positive correlation between the value of the maturity test conducted by the tutor and the test (0.17) (figure 1). In the variability assessment, the “ceiling effect” and the “ground effect” remain below 15 per cent (table 3). For construct validity, a comparison of test scores was carried out in relation to other recorded variables. Female participants obtained consistently higher scores both in the validated test as in the maturity assessment performed by the tutor. Regarding age, differences between scores were not significant. There were no significant differences regarding the type of school (public or subsidized) or origin (rural or urban) (table 4). Regarding the sensitivity to change, test–retest comparison, students showed an average improvement of 0.39 points (table 5).

## Discussion

### Methodological Discussion: Can the Maturity of A Minor Be Measured?

The present study aimed to design a valid, reliable, and easy-to-manage scale for assessing the maturity of adolescents. This scale was designed to be used in a clinical population given that no such scale meets these requirements at present.

Regarding psychometric characteristics, the examined test has a high feasibility rate, as it was completed by over 99 per cent of respondents. The estimated duration of T17 is 23.2 minutes on average (IC95 per cent: 22.5–23.9). For T9, the estimated duration is about 15 minutes. This calculation is based on an approach that considers the response time of each test item for the T9 test as proportional to the duration of the T17 test. However, the duration of completion and correction of the Kohlberg MJI or the Rest DIT is approximately 1.5 hours. The reliability, measuring internal consistency, obtained using the KR-20 remains around 0.5—a rating considered as moderate (Thorndike 1989). This moderation is explained in part by the design of the test: limited extension, dichotomous response structure, and multidimensionality (Ruiz Bolivar 2011). The moderation is also explained by the homogeneity in terms of age and culture of the population for which the test was performed. When compared with other tests measuring adolescents’ competence to decide, the Youth Decision-Making Competence (Y-DMC), a test consisting of seven subscales, two of which have dichotomous data and low number of items, obtains Alpha-CR coefficients of 0.30 and 0.03 (Bruine De Bruin, Fischhoff, and Parker 2007). In contrast, the Values in Action Inventory for Youth (VIA-Youth) obtains an Alpha-CR > 0.7, with a test of 198 items (Park and Peterson 2006). However, in a recent validation of the Rest DIT in a Turkish university population, an Alpha-CR of 0.5 was obtained (Cesur and Topçu 2010). The intraclass correlation coefficient (ICC) is 0.79 (T-17) and 0.77 (T-9), a high value, much better than that of VIA-Youth or others, with an ICC above 0.5.

Regarding structural validity, the variability of the test shows an appropriate discriminative capacity regarding the maturity of the participants (“ceiling and floor effect” < 15 per cent). Regarding convergent validity, the test has a high correlation with the gold standard used (T17: ICC=0.74/T9 ICC=0.65), positioning itself as a good test to measure the maturity of minors. In the study of the validity of extreme groups, it must be noted that regarding age, the data in the assessment of maturity by the tutor show a statistically significant progressive upward trend, something also seen in the test under validation and in the MJI, as expected (Chenneville et al. 2010; Moon 1986; Billick et al. 2001), although in these two cases, the high score of twelve-year-olds must be emphasized. In light of the reviewed literature, this suggests that twelve-year olds still work with a pattern of the values transmitted by their parents: this might explain why they take less time giving a mature response (Thurstone 1972; Marsh 1989).

In this line of thought, regarding informed consent, Grisso (1972) supported by Melton (1981) and Pearce (1994) suggest that it is towards fourteen years when a minor is able to give consent. Leikin (1983) argues that at age twelve, many children are already competent to make decisions, and Mann (1989) considers that at around age fifteen to seventeen, they achieve the adequate self-esteem necessary to make decisions. However, all children of school age highly value the fact of being informed of and being able to participate in decisions affecting their own health (Coad and Shaw 2008; Jeremic et al. 2016; Martenson and Fagerskiold 2007).

Therefore, their thoughts should always be taken into account before making any decision concerning them. Regarding gender, the test to validate, in line with the published literature, points to a more rapid development of maturity in adolescent girls (Cesur and Topçu 2010; Alderson 1993; Gibbs et al. 2007). Sensitivity to change is explored by observing whether the same approach of dilemmas can, over time, develop the moral competence of the individual. In this sense, there is an improvement with statistical significance in the test score of 0.3, supporting the idea that education in ethical conflicts, through reflection and discussion about moral dilemmas, is able to improve maturity in reasoning and decision-making (Ruhe et al. 2015).

### Conceptual Discussion

This test raises hypothetical ethical conflicts that classify maturity stages according to the preconventional or conventional moral stages of Kohlberg. The test results correlate with the maturity assessed by the gold standard (MJI) and with the appraisal made by the tutors that it is a useful tool to measure maturity.

To classify the degree of maturity of a minor based on the test, results were compared with the MJI stages, and three groups of maturity are proposed: a higher score corresponds to more mature individuals and vice versa (table 6).

**Table 6:** Maturity T9 test ranges proposal, according to Kohlberg’s maturity stages

	Stage		
T9 test	I-II	II-III	III-IV
<= 5	76 %	25	0 %
6-7	21 %	37	16 %
8-9	3%	38	84 %

However, the minor’s maturity is an important part of the competence assessment, but not the only one. Revising the literature, we propose a structured and multidimensional way of assessing children’s decision-making competence, with four complementary areas: evaluation of the maturity of moral reasoning of the minor, assessment of the minor's ability to understand information, assessment of the gravity of the decision, and assessment of the minor's context and of the decision (Pearce 1994; Leikin 1983; Mann, Harmony, and Power 1989).

In the second step, evaluating the ability to understand the provided information, Appelbaum (2007) suggest the following four criteria of assessment in adults: understanding of the relevant information to make a decision, appreciation of the situation and its consequences, rational manipulation of information, and ability to make a choice. They developed the MacArthur Assessment Tool, with different version for treatment or clinical research.

In minors, a noteworthy study was realized by Weithorn and Campbell (1982), in which Appelbaum’s criteria were applied in a group of healthy minors and adolescents (aged nine, fourteen, eighteen and twenty-one), exposing them to four hypothetical dilemmas involving healthcare decisions. All four standards of competence (ability of choice, rational choice, rational motivation, understanding of the decision) were assessed. All children under the age of fourteen decided in a similar manner to young people aged from eighteen to twenty-one. Other studies applied the MacArthur Assessment Tool in clinical adolescent population, with mixed results (Miller, Drotar, and Kodish 2004; Koelch et al. 2010; Turrell, Peterson-Badali, and Katzman 2011).

In the third step, it’s also important to consider the gravity of the minor’s decision. Drane’s Sliding Scale of Competency (1994) refers to the proportionality of the decision (the more serious the decision, the greater the level of competence required from the person taking it). It is the benchmark for this type of assessment.

And in the fourth step, other factors that influence the degree of involvement of adolescents in decisions must also be taken into consideration (McCabe et al. 1996; Marsh 1989; Reder and Fitzpatrick 1998), whether they depend on the minor themselves (pain, anxiety, consumption of medicines or drugs) or on family or cultural issues and situational factors (doctor–patient relationship of trust, emergency context) (McCabe et al. 1996; Chenneville et al. 2010; Pearce 1994; Mann, Harmony, and Power 1989; Reder and Fitzpatrick 1998; Shaw 2001).

Among health professionals in their usual clinical practice, this tool offers a reflection on the minor's participation in a daily consultation and on their ability to make decisions regarding their own health, simply because they have a tool designed to assist them in the assessment of this competence. The physician needs to acquire certain skills to fulfil this task (Martenson and Fagerskiold 2007).

Furthermore, facilitating communication between children, parents, and professionals and involving children in the process of decision-making provides greater satisfaction regarding the provided medical care, both for parents and children, enhanced cooperation from the child during treatment, and an increased sense of control, resulting in the illness being perceived as less stressful and reducing the feeling of discomfort; not to mention it also shows respect for the child’s abilities and encourages their development (McCabe et al. 1996; Alderson 1993).

The limitations of the study include the structure of the test. Its dichotomous nature, multidimensionality, and short length may be limiting factors in the degree of reliability achieved, although the short length was needed to design a quick and easy-to-use tool. Another element that can influence the outcome of this coefficient is that the study population was very homogeneous; the tool also needs to be tested in heterogeneous environments. There is no proper gold standard, despite using an indirect, semiqualitative measurement through the MJI and the evaluation of the tutor. The test still needs validation in the real context of healthcare decisions.

## Conclusions and Future Prospects

Moral competence is a process that is gradually acquired by an individual. Moral competence is the basis of the minor’s maturity and is necessary for assessing the minor’s competence in health decision-making.

Following the main objective of this study, a test of reasoning on moral conflicts was designed and validated to infer the degree of maturity of the interviewed minor. Three stages of maturity have been established in accordance with the stages of moral judgment proposed by Kohlberg.

The assessment of the content validity and test criteria showed a high correlation with the score obtained with the gold standard, MJI, and with the maturity assessment of the tutor. A moderate reliability coefficient (≈0.45) with a high CCI (> 0.6) was also obtained.

Light has been shed on the influence of age, gender, environment, and education—among other factors—on the development of maturity. Especially noteworthy was that females seem to develop their maturity earlier at this age.

These results indicate that this is a useful, objective, rapid, and reliable tool for measuring the maturity of minors through reflection on ethical issues.

This tool needs to be tested in a clinical environment to confirm its validity in particularly vulnerable situations, to explore how illness and associated emotional distress affect maturity, and to compare it with other tools used in such situations.

## Appendix 1

### MADURTEST (T9)

**Case 1:** You have just bought a computer on Internet. When you did so, you clicked on the option “send money trough post”, but in the end, you forgot to pay. When you get the computer, it has a stick saying”PAYED”. What would you do?

1. I inform that I haven't played yet, and I pay, so it's not good to cheat.
2. I call them and pay, because they could find out the mistake, and make me pay more money.
3. I don't say anything, and if they call me, I would go off the track.
4. I don't inform, so the mistake is not my responsibility.

**Case 2:** Maria is a single mother with two little child in charge. She's go a neighbour with AIDS; they are good friends. One day Maria listen to his neighbour's shouting, and she finds him bleeding through a deep wound. Maria calls the ambulance which can be there for about 15 minutes, and ask her to stop up the wound. Maria hasn't got any gloves and she knows about the contagiousness of AIDS.

1. She should help him, other ways he will die.
2. She shouldn't help him because he's got AIDS.
3. She should help him because if she let him die she can be into trouble.
4. She shouldn't help him because noon is obliged to help when there's a big danger.

**Case 3:** Julian has a friend who stoles a motorbike and brakes it. Julian promises his friend he won't explain anybody. But one day he realizes another boy has been accused and he's going to jail. Julian asks his friend to confess the theft but he answers negatively and reminds him of his promise. If Julian doesn't confess, an innocent is going to be accused, but if he confesses, he'll break a promise to his friend.

1. He shouldn't confess, because a promise to a friend is above all.
2. He shouldn't confess, because he's going to be in trouble.
3. He should confess, because if anybody realizes Julian can also be blamed.
4. He should confess because otherwise an innocent will be damaged.

**Case4:** Dr. Martinez is testing a new vaccine that can save many people all through the world. This vaccine is in good progress, there's only left the last phase in which he must try between two excipients. If he is wrong he will causes patient's death wile experimenting, but this will let him get the right vaccine. The doctor has a possibility to experiment with condemned people so they can reduce their condemn.

1. He should experiment so he can achieve success and scientific prestige.
2. He shouldn't experiment because condemned people are not free to decide.
3. He should experiment because society's benefit obtained is big.
4. He shouldn't experiment because if a condemned person dies, he can be reported.

**Case 5:** José has friend with a degenerative illness that makes him paralytic progressively. His friend shows him an envelope and tells him that there is a poison inside. He asks José to give it to him when he can't move at all.

1. He should accept to reduce his friend's suffering.
2. He shouldn't accept because he can be into trouble.
3. He shouldn't accept because it is forbidden by law.
4. He should accept to help his friend, and nobody can realize.

**Case 6:** Juan has a good friend called Diego. Diego consume cocaine and sometimes invites Juan, but he doesn’t want to try so. One day the headmaster of the school calls Juan and tells him that he knows that a friend of him consumes cocaine and asks him his name. If he doesn’t help the school, it can be considered a harmful behaviour for the school and can be driven out.

1. He should tell the truth because Diego is doing wrong consuming cocaine.
2. He should tell the truth to avoid the punishment.
3. He shouldn’t tell the truth because friendship is above all.
4. He shouldn’t tell the truth because a friend cannot be betrayed, because “he will help me another time, these are friends”

**Case 7:** You are having some trouble with your couple. He asks you about a friend of yours at your school. He only asks you about that to feel better, not being suspicious. But you are more than just friend with yours school mate. What would you do?

1. I tell him the truth, because a love my couple, I respect him, and this is not fair to him.
2. I tell him it's ok, that he's the only one in my life, because soon I'll be bored with my friend and I love my couple.
3. I tell him I only love him, while I think how I can get out of this mess.
4. I tell him the truth, because I wouldn't like to be deceived.

**Case 8:** At the outskirts of your village, there's a big and rich company that trough toxic residuum to the river without permission. You belong to an ONG that fights for a more ecologic and better world; you organize a pacific demonstration, but it's not important on communication media. You think about an alternative “civil disorder”: causing damage to the company, so your protest can be heard. ¿Is it justified to respond badly to the bad behaviour?

1. Yes, it's for a good reason.
2. No, never the aim justifies any way.
3. Yes, because is the only way to make us be heard.
4. No, because the reprimand can be worse.

**Case 9.**your best friend is very sad, lately. She explains to you that a familiar of her has abuse of her. She doesn't want anybody to know about it because she's ashamed and thinks of suicide if people know about that. ¿What would you do?

1. I would explain it to her parents because my friend is suffering a lot and needs some help.
2. I would explain it to her parents because a person has made a mistake and must be in jail.
3. I wouldn't explain it because I want to respect her wish.
4. I wouldn't explain it because is none of my business...
